# Supplementation of essential amino acids suppresses age-associated sleep loss and sleep fragmentation but not loss of rhythm strength under yeast-restricted malnutrition in *Drosophila*

**DOI:** 10.1093/jb/mvae090

**Published:** 2024-12-19

**Authors:** Sachie Chikamatsu, Yasufumi Sakakibara, Kimi Takei, Risa Nishijima, Koichi M Iijima, Michiko Sekiya

**Affiliations:** Department of Neurogenetics, Center for Development of Advanced Medicine for Dementia, National Center for Geriatrics and Gerontology, 7-430 Morioka-cho, Obu, Aichi 474-8511, Japan; Department of Experimental Gerontology, Graduate School of Pharmaceutical Sciences, Nagoya City University, 3-1 Tanabe-dori, Mizuho-ku, Nagoya, Aichi 467-8603 Japan; Department of Neurogenetics, Center for Development of Advanced Medicine for Dementia, National Center for Geriatrics and Gerontology, 7-430 Morioka-cho, Obu, Aichi 474-8511, Japan; Department of Neurogenetics, Center for Development of Advanced Medicine for Dementia, National Center for Geriatrics and Gerontology, 7-430 Morioka-cho, Obu, Aichi 474-8511, Japan; Department of Neurogenetics, Center for Development of Advanced Medicine for Dementia, National Center for Geriatrics and Gerontology, 7-430 Morioka-cho, Obu, Aichi 474-8511, Japan; Department of Neurogenetics, Center for Development of Advanced Medicine for Dementia, National Center for Geriatrics and Gerontology, 7-430 Morioka-cho, Obu, Aichi 474-8511, Japan; Department of Experimental Gerontology, Graduate School of Pharmaceutical Sciences, Nagoya City University, 3-1 Tanabe-dori, Mizuho-ku, Nagoya, Aichi 467-8603 Japan; Department of Neurogenetics, Center for Development of Advanced Medicine for Dementia, National Center for Geriatrics and Gerontology, 7-430 Morioka-cho, Obu, Aichi 474-8511, Japan; Department of Experimental Gerontology, Graduate School of Pharmaceutical Sciences, Nagoya City University, 3-1 Tanabe-dori, Mizuho-ku, Nagoya, Aichi 467-8603 Japan

**Keywords:** Drosophila, sleep, malnutrition, ageing, amino acid

## Abstract

Sleep quality and quantity decrease with age, and sleep disturbance increases the risk of many age-associated diseases. There is a significant relationship between nutritional status and sleep outcomes, with malnutrition inducing poor sleep quality in older adults. However, it remains elusive whether, and if so how, nutritional supplementation prevents age-associated sleep problems. Here, we utilized *Drosophila* to investigate the effects of a malnutrition diet with restricted yeast, a primary protein source, and supplementation of 10 essential amino acids (EAAs) on sleep profiles during ageing. Compared with the standard diet containing 2.7% yeast, the malnutrition diet containing 0.27% yeast significantly decreased target of rapamycin (TOR) signalling and shortened the lifespan of male Canton-S flies. By contrast, age-associated sleep loss, sleep fragmentation and loss of rhythm strength were similarly observed under both diets. Supplementation of the malnutrition diet with EAAs in restricted yeast significantly ameliorated age-associated sleep loss and sleep fragmentation without altering loss of rhythm strength. It also rescued decreased TOR signalling activity but not the shortened lifespan, suggesting that the effects of EAAs on sleep integrity are independent of TOR activity and lifespan regulation. These results may help to develop dietary interventions that improve age-related sleep problems in humans.

## Graphical Abstract

**Graphical Abstract:**
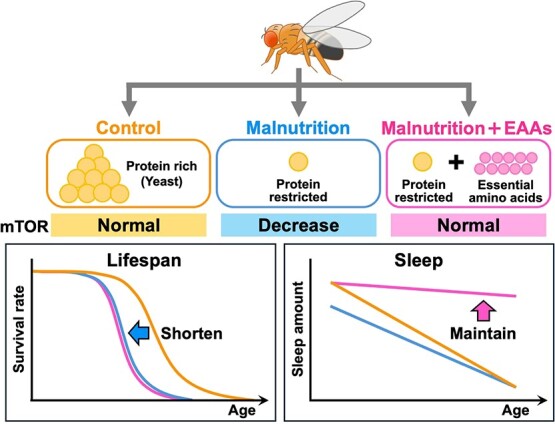

Caloric restriction, including intermittent fasting and dietary restriction without malnutrition, delays ageing and extends lifespan in multiple model organisms, including primates (1). In humans, mild (14%) caloric restriction positively impacts health span, including reduced levels of ectopic lipids and enhanced thymic function in middle age (2). However, severe caloric restriction negatively affects growth, reproduction and immune defence in return for longevity (3).

Older adults are prone to undernutrition (referred to as malnutrition) due to altered tastes as well as environmental changes caused by physical disability or disease (4, 5). Poor nutrition status leads to declines in multiple physiological functions, such as mobility, balance, muscle strength, motor processing, cognition, endurance and physical activity (6), and predisposes older adults to an increased risk of mortality and morbidity (7, 8). Thus, a well-balanced diet without malnutrition is important for both health and lifespan benefits.

Sleep is a physiological phenomenon observed across various species (9, 10). Although the function of sleep is not fully understood, it serves multiple purposes, such as homeostatic support of the cardiovascular system, immune system and memory, as well as clearance of waste products from the brain (11–16). Sleep quality and quantity decrease as we age, and older adults have an increased prevalence of sleep disorders such as insomnia (17). Disruption of the regular sleep architecture is a frequent antecedent to the onset of dementia (18, 19), and an association between sleep duration and cognitive impairment has also been reported (20). In addition, epidemiological studies suggest that inadequate sleep duration and poor sleep quality are associated with an increased risk and higher incidence of cardiovascular and metabolic diseases including coronary artery disease, stroke and diabetes (11, 21–24). Moreover, sleep disturbance has been associated with dysregulated immune responses, thus contributing to an increased risk of infection and inflammation-related chronic diseases (12, 25). Thus, elucidation of the mechanisms underlying age-associated sleep problems is critical to prevent many age-related diseases.

There is a significant relationship between nutritional status and sleep outcomes (26), and malnutrition has been associated with poor sleep quality in older adults (27–30). In addition, sleep is influenced by dietary factors, such as fat, sugar and amino acids (31, 32). However, in human studies, it is difficult to strictly control the nutritional status and examine the direct effects of nutritional conditions on sleep during ageing. It remains elusive whether, and if so how, nutritional supplementation prevents age-associated sleep problems.

For >100 years, *Drosophila melanogaster* has been an excellent model organism not only for genetics and developmental biology but also for neuroscience, metabolism, ageing and human disease modelling (33, 34). Approximately 75% of genes related to human diseases are shared in *Drosophila* (35), and dietary restriction extends lifespan in adult flies (36). Sleep research in *Drosophila* is relatively new and was initiated by two studies published in 2000 (37, 38). These studies showed that the sleep-like state in *Drosophila* has the same characteristics as sleep in mammals: 1) behavioural quiescence (sleep-like state), 2) increased arousal threshold, 3) rapid reversibility with a sufficiently strong stimulus, 4) increased sleep after sleep deprivation (rebound) and 5) response to drugs acting on human sleep and arousal (10, 37–39). Of particular importance, sleep loss, sleep fragmentation and loss of rhythm strength have been observed in aged *Drosophila* (38, 40, 41), suggesting that *Drosophila* is an excellent model to study the mechanisms underlying sleep disruption during ageing.

In this study, we systematically investigated the effects of a malnutrition diet and supplementation of essential amino acids (EAAs) on age-associated alterations of sleep profiles in *Drosophila*.

## Materials and Methods

### Fly stocks and maintenance

Flies were maintained in the standard diet (see Table 1) (42) on a 12:12-h light:dark cycle at 25°C. Canton-S (BDSC #64349) flies were obtained from the Bloomington *Drosophila* Stock Center.

**Table 1:** Comparison of the composition of *Drosophila* medium (diets) used in the fly stock centres and our laboratory

**Ingredient**	**This study**	**BDSC**	**VDRC**	**Kyoto**
Agar (g/l)	6.4	5.3	7.6	7.2
Yeast (g/l)	27.0	15.9	17.1	40
Soy flour (g/l)		9.2	9.5	
Corn meal (g/l)	64.3	67.1	76.0	
Corn flour (g/l)				46.7
Corn grits (g/l)				23.3
Sucrose (g/l)	26.5			
Glucose (g/l)	53.0			100
Corn syrup (ml/l)		70.6		
Molasses (ml/l)			22.0	
Moult extract (ml/l)			76.0	
Potassium tartrate (g/l)	7.4			
Calcium chloride (g/l)	0.6			
Propionic acid (ml/l)	4.5	4.4	8.1	5.0
Phosphoric acid (ml/l)	0.5		0.5	
Nipagin (g/l) (methyl parahydroxybenzoate)	0.7		1.7	0.5

BDSC, Bloomington *Drosophila* Stock Center; Kyoto, Kyoto *Drosophila* Stock Center; VDRC, Vienna *Drosophila* Resource Center.

### Fly media

The standard diet containing 6.4% cornmeal, 2.7% sucrose, 5.3% glucose, 2.7% yeast and 0.6% agar (Table 1) was prepared as previously described (42). The malnutrition diet was prepared in the same way as the standard diet but with 0.27% yeast instead of 2.7% yeast. To prepare the malnutrition + EAAs diet, EAAs were added to the malnutrition diet in order to achieve the same concentrations of amino acids present in the yeast component of the standard diet. The composition of amino acids in the diets used in this study is shown in Table 2. The amino acids used in this study were lysine (Tokyo Chemical Industry, Cat#. L0129), leucine (Nacalai Tesque, Cat#. 20,327–62), phenylalanine (Nacalai Tesque, Cat#. 26,910–22), tryptophan (Nacalai Tesque, Cat#. 35,607–32), histidine (Nacalai Tesque, Cat#. 18,116–92), isoleucine (Nacalai Tesque, Cat#. 20,330–02), methionine (Kanto Chemical, Cat#. 25,613–30), threonine (Nacalai Tesque, Cat#. 33,820–82), valine (Nacalai Tesque, Cat#. 36,108–42) and arginine (Nacalai Tesque, Cat#. 03321–52).

**Table 2:** Amino acid composition in the standard diet, malnutrition diet and malnutrition + EAAs diet

**Amino acid contents (g/l)**	**Standard**	**Malnutrition**	**Malnutrition**	**+EAAs**
**Yeast**	(27.0)	(2.7)	(2.7)	
Alanine	0.97	0.10	0.10	
**Arginine**	0.78	0.08	0.08	0.70
Aspartate	1.43	0.14	0.14	
Cystine	0.14	0.01	0.01	
Glutamate	1.70	0.17	0.17	
Glycine	0.62	0.06	0.06	
**Histidine**	0.35	0.04	0.04	0.32
**Isoleucine**	0.65	0.06	0.06	0.58
**Leucine**	1.00	0.10	0.10	0.90
**Lysine**	1.11	0.11	0.11	1.00
**Methionine**	0.24	0.02	0.02	0.22
**Phenylalanine**	0.59	0.06	0.06	0.53
Proline	0.51	0.05	0.05	
Serine	0.76	0.08	0.08	
**Threonine**	0.73	0.07	0.07	0.66
**Tryptophan**	0.19	0.02	0.02	0.17
Tyrosine	0.43	0.04	0.04	
**Valine**	0.78	0.08	0.08	0.70

Ten essential amino acids for *Drosophila* are in bold.

### Fly experiments

Flies were raised in standard cornmeal media. After eclosion, male flies were collected at 0–1 day old, and 25 flies were placed in a vial containing the standard diet (Control), malnutrition diet (Malnutrition) or malnutrition diet with EAAs (Malnutrition + EAAs). The vials were replaced with fresh ones every 3 or 4 days, and the flies were maintained until they were used for each experiment.

### Lifespan analysis

Lifespan was analysed as described previously (43). Food vials containing 25 male flies were placed sideways at 25°C in 60% humidity on a 12:12-h light:dark cycle. Food vials were changed every 2 or 3 days, and the number of dead flies was counted each time. At least six vials per diet were prepared.

### Sleep and activity analysis

Male flies were placed individually in glass tubes (length, 65 mm; diameter, 5 mm) containing fly food at one end and a cotton plug at the other end. The glass tubes were set up on *Drosophila* activity monitors (DAMs) (DAM2, Trikinetics) and maintained under 12:12-h light:dark cycle at 25°C. The number of infrared beam crossings was recorded every 1 min and collected using DAMsystem3 (Trikinetics). Data from two consecutive days, at least 1 day after the setup, were used for subsequent analyses. Raw data files were analysed with DAMfilescan113X (Trikinetics), and sleep was defined as a period of inactivity lasting 5 min (37, 38). Total activity counts, sleep amount and other parameters were analysed using Microsoft Excel (Microsoft). Fast Fourier transform values were calculated using ActogramJ (https://bene51.github.io/ActogramJ/index.html).

### Western blotting

Western blotting was performed as described previously (43, 44). More than 10 fly heads per diet were homogenized in Tris-glycine SDS sample buffer (Thermo Fisher, Cat#. LC2676), and the same amount of lysate was loaded in each lane of Tris-glycine gels and transferred to nitrocellulose membranes. The membranes were blocked with 5% nonfat dry milk, blotted with primary antibodies described below, incubated with appropriate secondary antibodies and developed using ECL Prime Western Blotting Detection Reagent (Cytiva). The membranes were also probed with an anti-tubulin antibody, which was used as the loading control for each experiment. Rabbit polyclonal anti-phospho-*Drosophila* p70 S6K (Thr398) (Cell Signaling Technology, Cat#. 9209; 1/1000), sheep polyclonal anti-*Drosophila* p70 S6K (MRCPPU Reagents, S045B, 1/500) (45) and mouse monoclonal anti-α-tubulin (Sigma-Aldrich, Cat#. T9026; 1/100,000) antibodies were used for immunoblotting. Horseradish peroxidase-conjugated anti-mouse IgG (Cytiva, Cat#. NA931-1ML; 1/4000), anti-rabbit IgG (Cell Signaling Technology, Cat#. 7074; 1/2000) and anti-sheep IgG (R&D Systems, Cat#. HAF016; 1/1000) were used as secondary antibodies. Imaging was performed with Amersham Imager 680 (Cytiva), and the signal intensity was quantified using ImageJ (NIH).

### Statistical analysis

All results are expressed as mean ± standard error of the mean (SEM). The unpaired Student’s *t*-test, a one-way analysis of variance (ANOVA) with Tukey’s *post hoc* test, and Kaplan–Meier survival analyses with log-rank tests (GraphPad Prism 10, GraphPad Software) were used to determine statistical significance as indicated in the figure legends. ^*^ indicates *P* < 0.05, ^**^ indicates *P* < 0.01, and ^***^ indicates *P* < 0.001.

## Results

### The standard and malnutrition diets used in this study

Dietary restriction experiments in *Drosophila* often restrict the amount of yeast, a major source of protein in fly food (46). Each laboratory has their own fly food recipe; therefore, we first compared the composition of fly food used in our laboratory with those used in three major *Drosophila* stock centres (Table 1). Although each fly food includes slightly different ingredients, all contain similar levels of nutrition, including 2.5–4% yeast or yeast plus soy flour as a protein source (Table 1). In this study, we defined our fly food containing 2.7% yeast as the standard diet (Control) and fly food containing 0.27% yeast as the malnutrition diet (Malnutrition).

### Age-associated alterations of sleep profiles under the standard diet in *Drosophila*

**Fig. 1:**
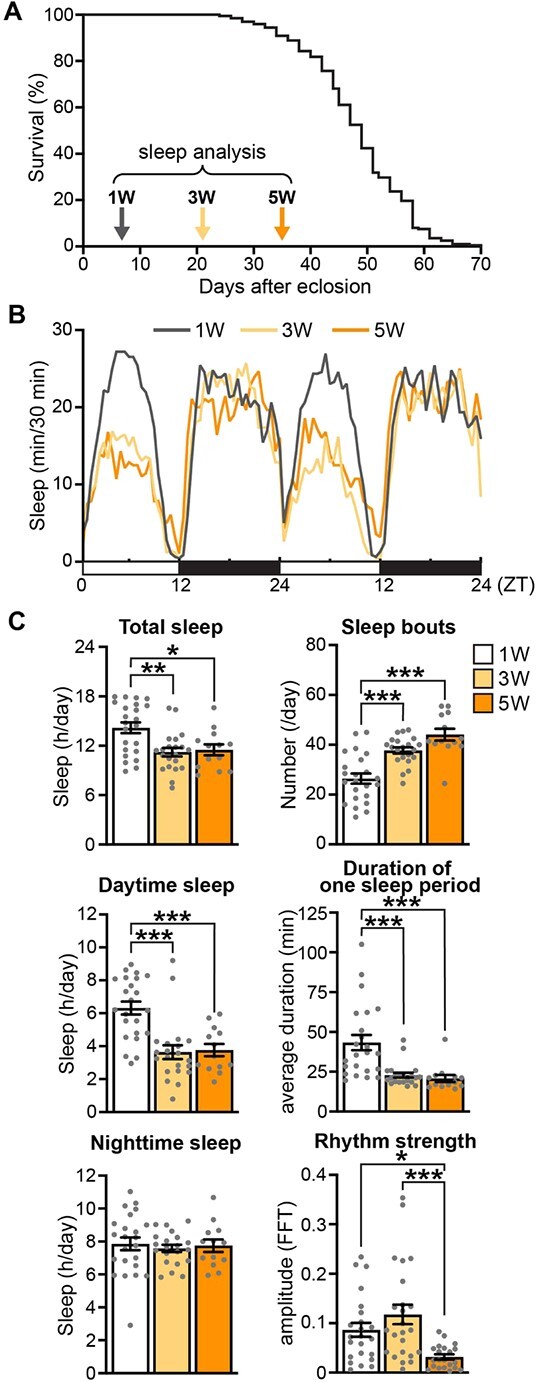
**Age-associated alterations of sleep profiles under the standard diet in *Drosophila.*** (A) The lifespan of wild-type (Canton-S) male flies maintained on the standard diet. *n* = 198; median lifespan, 49 days; maximum lifespan, 70 days. Sleep analyses were performed at the age of 1, 3 and 5 weeks (indicated by arrows). (B) Sleep profiles of wild-type male flies at the age of 1, 3 and 5 weeks. Average sleep amounts in the 30-min bin are plotted at Zeitgeber time (ZT). Daytime and night-time under the light–dark condition is indicated in white and black bars at the bottom. *n* = 23, 1 week of age (1 W); *n* = 21, 3 weeks of age (3 W); *n* = 13, 5 weeks of age (5 W). (C) Total sleep, daytime sleep, night-time sleep, number of sleep bouts, duration of one sleep period and rhythm strength are shown as mean ± SEM. *n* = 13–23, ^*^P < 0.05, ^**^P < 0.01 and ^***^P < 0.001 by one-way ANOVA followed by Tukey’s *post hoc* tests.

To examine the effects of diets on age-associated alterations of sleep profiles in *Drosophila*, we employed the wild-type Canton-S strain. To exclude any trade-off effects resulting from fecundity (egg laying) in female flies, we used male flies for the analysis. We first examined the survival rate of male Canton-S flies under the standard diet. Flies were raised in the standard diet, adult male flies were collected after eclosion and maintained on the standard diet *ad libitum* and survival rates were determined. The median and maximum lifespans of male Canton-S flies were 49 and 70 days, respectively (*n* = 198, Fig. 1A).

**Fig. 2:**
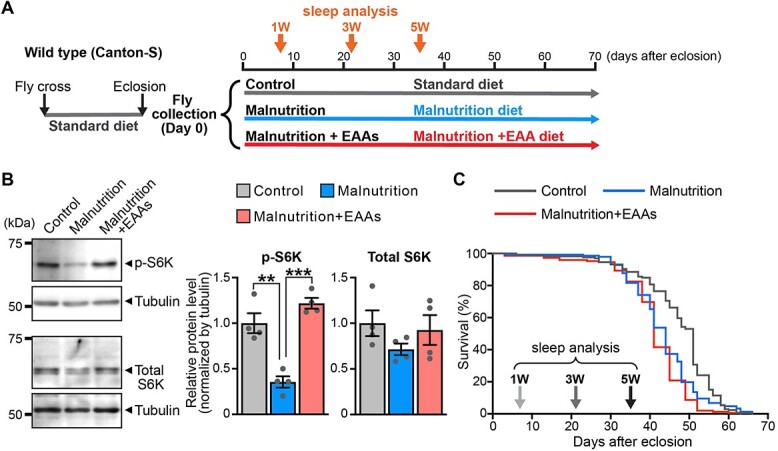
**The yeast-restricted malnutrition diet decreases TOR signalling and shortens the lifespan of flies, whilst EAAs supplementation rescues decreased TOR signalling activity but not shortened lifespan under the yeast-restricted malnutrition diet.** (A) Schematic representation of fly experiments. Flies were raised in standard diet. After eclosion, male flies were collected and placed in each vial containing standard diet (Control), malnutrition diet (Malnutrition) or malnutrition diet with EAAs (Malnutrition + EAAs). Sleep analysis, western blot analysis and survival assay were performed under each diet condition. (B) Levels of phosphorylated S6 kinase (p-S6K) were reduced in flies maintained on the yeast-restricted malnutrition diet and were restored by the addition of EAAs. Quantifications of p-S6K and total S6K protein levels are normalized by tubulin and shown on the right. Mean ± SEM, *n* = 4 technical replicates, ^**^P < 0.01 and ^***^P < 0.001 by Student’s *t*-test. (C) The lifespan of male wild type flies maintained on the standard diet (Control), malnutrition diet (Malnutrition) or malnutrition diet supplemented with EAAs (Malnutrition + EAAs). The lifespans were determined by Kaplan–Meier survival analysis with log-rank test. *P* < 0.001; Control versus Malnutrition, *P* < 0.001; Control versus Malnutrition + EAAs; not significant; Malnutrition versus Malnutrition + EAAs. Control, *n* = 166; median lifespan, 51 days; maximum lifespan, 66 days; Malnutrition, *n* = 147; median lifespan, 44 days; maximum lifespan, 66 days; Malnutrition + EAAs, *n* = 149; median lifespan, 41 days; maximum lifespan, 63 days.

Wild-type Canton-S flies exhibit age-associated alterations of sleep profiles including sleep loss, sleep fragmentation and loss of rhythm strength (37, 38, 40, 41). We examined the sleep profiles of male flies at 1, 3 and 5 weeks of age before the survival rate declined under the standard diet (Fig. 1A, time points indicated by arrows). Using DAMs, we analysed locomotor activity and sleep profiles during two consecutive days under a 12:12-h light:dark cycle. We considered the absence of locomotor activity for >5 min as sleep because flies that had been behaviourally quiescent for ≥5 min rarely showed a behavioural response to stimuli that behaviourally awake flies readily responded to (38). Consistent with a previous report (40), the amount of daytime sleep decreased upon ageing in male Canton-S flies (Fig. 1B). Quantitative analysis revealed that total sleep and daytime sleep were significantly lower in 3- and 5-week-old flies than in 1-week-old flies (Fig. 1C, see Total sleep and Daytime sleep). In addition, the number of sleep bouts was significantly higher and the average duration of one sleep period was significantly lower in 3- and 5-week-old flies than in 1-week-old flies, indicating that sleep fragmentation occurred during ageing (Fig. 1C, see Sleep bouts and Duration of one sleep period). Moreover, the rhythm strength of the sleep:wake cycle was significantly lower in 5-week-old flies than in 1- and 3-week-old flies (Fig. 1C, see Rhythm strength). The waking activity, which represents the level of locomotor activity, and the duration of one activity period were significantly decreased in 5-week-old flies, indicating that age-associated sleep loss was unlikely due to increased activity in aged flies (Supplementary Fig. S1).

In summary, these data confirmed that age-associated alterations of sleep profiles, including sleep loss, sleep fragmentation and loss of rhythm strength in adult male Canton-S flies, can be investigated under our standard diet and experimental conditions.

### The yeast-restricted malnutrition diet decreases target of rapamycin signalling and shortens the lifespan of flies

To examine the effects of yeast-restricted malnutrition on age-associated alterations of sleep profiles, we first examined the lifespan of male Canton-S flies under the malnutrition diet. Flies were raised in the standard diet during development, and adult male flies were collected after eclosion and maintained on the malnutrition diet (Malnutrition) or standard diet (Control) *ad libitum* (Fig. 2A). The target of rapamycin (TOR) signalling pathway is activated in response to an availability of amino acids, and TORC1, a component of TOR signalling, regulates translation and growth by phosphorylating key downstream effectors, including ribosomal S6 kinase (S6K).

As expected, the phosphorylation level of S6K (p-S6K) was significantly lower in flies maintained on the malnutrition diet than in flies maintained on the standard diet (Fig. 2B, Control vs Malnutrition). The total protein level of S6K was also slightly lower in flies maintained on the malnutrition diet, but the difference did not reach statistical significance. Under these conditions, flies maintained on the malnutrition diet (Malnutrition) had slightly but significantly shorter lifespans than flies maintained on the standard diet (Control) (Fig. 2C, median lifespan of Control, 51 days; median lifespan of Malnutrition, 44 days, *P* < 0.001 by the log-rank test).

### EAA supplementation rescues decreased TOR signalling but not the shortened lifespan under the yeast-restricted malnutrition diet

Yeast is a major source of protein in fly food; therefore, we next examined whether supplementation of the malnutrition diet with 10 amino acids essential for *Drosophila* (EAAs) (47) rescued the decreased TOR signalling and shortened lifespan of flies. To achieve the same concentration of EAAs as in the standard diet, we calculated the level of each EAA in yeast in the malnutrition diet compared with the standard diet, and each EAA was added back to the malnutrition diet accordingly (Malnutrition + EAAs) (Table 2).

Flies were raised in the standard diet, and adult male flies were collected after eclosion and maintained on the malnutrition diet supplemented with EAAs (Malnutrition + EAAs) *ad libitum* (Fig. 2A). As expected, supplementation of EAAs rescued the decreased phosphorylation level of S6K (p-S6K) in flies maintained on the malnutrition diet (Fig. 2B, top panels, Malnutrition vs Malnutrition + EAAs, quantification, right). Supplementation of EAAs slightly increased the total protein level of S6K, but the difference did not reach statistical significance (Fig. 2B, bottom panels, Malnutrition vs Malnutrition + EAAs, quantification, right). However, supplementation of EAAs did not rescue the shortened lifespan of flies maintained on the malnutrition diet (Fig. 2C, Malnutrition vs Malnutrition + EAAs, median lifespan of Malnutrition, 44 days; median lifespan of Malnutrition + EAAs, 42 days, not significant by the log-rank test).

These results indicate that other nutrients contained in yeast in addition to EAAs, such as vitamins, lipids and dietary fibres, are required to rescue the shortened lifespan of flies on our malnutrition diet because vitamins and lipids affect the lifespan of flies.

### The yeast-restricted malnutrition diet slightly accelerates sleep loss in young flies

**Fig. 3:**
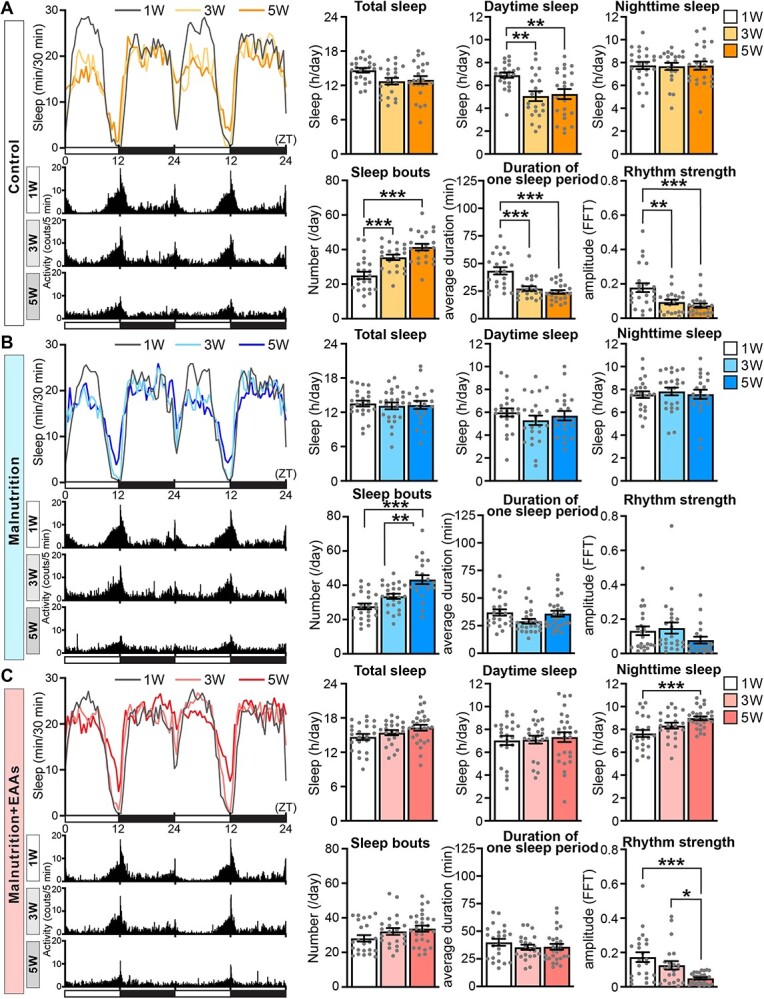
**Age-associated alterations of sleep profiles under the standard diet, yeast-restricted malnutrition diet and yeast-restricted malnutrition diet with EAAs in *Drosophila.*** Sleep profiles and actograms from wild-type Canton-S male flies maintained on the standard diet (Control) (A), yeast-restricted malnutrition diet (Malnutrition) (B) and malnutrition diet supplemented with 10 EAAs (Malnutrition + EAAs) (C) at the age of 1, 3 and 5 weeks. Average sleep amounts in the 30-min bin are plotted at ZT. Daytime and night-time under the light–dark conditions are indicated in white and black bars at the bottom. The actogram shows the average number of infrared beam crossings for 5-min-bin. Total sleep, daytime sleep, night-time sleep, number of sleep bouts, duration of one sleep period, and rhythm strength are shown as mean ± SEM. *n* = 21–24, ^*^P < 0.05, ^**^P < 0.01 and ^***^P < 0.001 by one-way ANOVA followed by the Tukey’s *post hoc* tests.

We next examined whether the malnutrition diet affected age-associated alterations of sleep profiles. Flies were raised in the standard diet, adult male flies were collected after eclosion and maintained on the standard diet (Control) or malnutrition diet (Malnutrition) *ad libitum* and sleep profiles were examined at 1, 3 and 5 weeks of age before the survival rate prominently declined under these diets (Fig. 3). Under the standard diet, daytime sleep decreased (sleep loss), the number of sleep bouts increased, the average duration of one sleep period decreased (sleep fragmentation), rhythm strength decreased (loss of rhythm strength) and activity, the waking activity, and the duration of one activity period decreased (activity loss) in 3- and/or 5-week-old flies similar to the data presented in Fig. 1 and Supplementary Fig. S1 (Fig. 3A and Supplementary Fig. S2A).

By contrast, under the malnutrition diet, age-associated alterations of sleep profiles slightly changed. The number of sleep bouts increased, and the waking activity and duration of one activity period decreased, suggesting that sleep fragmentation and activity loss occurred in 3- and/or 5-week-old flies compared with 1-week-old flies (Fig. 3B and Supplementary Fig. S2B). However, age-associated sleep loss and loss of rhythm strength were not evident under the malnutrition diet.

**Fig. 4:**
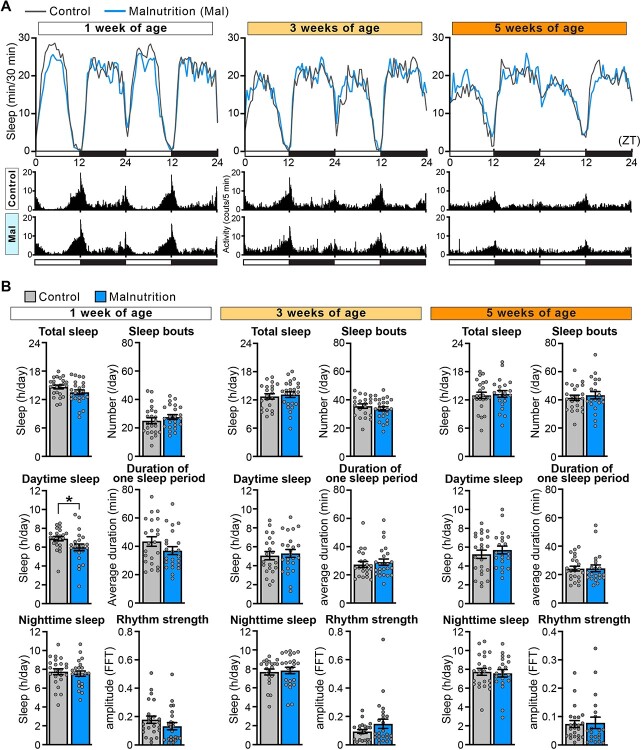
**The yeast-restricted malnutrition diet slightly accelerates sleep loss in young flies.** (A) Sleep profiles and actograms from wild-type male flies maintained on the standard diet (Control) or yeast-restricted malnutrition diet (Malnutrition) at the age of 1, 3 and 5 weeks. Average sleep amounts in the 30-min bin are plotted at ZT. Daytime and night-time under the light-dark conditions are indicated in white and black bars at the bottom. The actogram shows the average number of infrared beam crossings for 5-min-bin. (B) Total sleep, daytime sleep, night-time sleep, number of sleep bouts, duration of one sleep period and rhythm strength are shown as mean ± SEM. *n* = 21–28, ^*^P < 0.05 by Student’s *t*-test.

To further investigate the difference in sleep profiles under the standard and malnutrition diets, we directly compared these two datasets at each age (Fig. 4 and Supplementary Fig. S3). This analysis did not detect difference in any sleep parameter between the two groups except for the daytime sleep amount at 1 week of age, which was slightly but significantly lower under the malnutrition diet than under the standard diet (Fig. 4B, Daytime sleep at 1 week of age). These data suggest that malnutrition slightly accelerates age-associated sleep loss in flies and thus sleep amount did not further decrease after 1 week of age under the malnutrition diet (Fig. 3B). Rhythm strength was also slightly lower under the malnutrition diet at 1 week of age, but the difference did not reach statistical significance (Fig. 4B, Rhythm strength at 1 week of age).

### Supplementation of the malnutrition diet with EAAs suppresses age-related sleep loss and sleep fragmentation but not loss of rhythm strength and activity

We examined if supplementation of the malnutrition diet with EAAs to the same level as the standard diet increased the amount of daytime sleep at 1 week of age (Fig. 4B) and restored age-associated alterations of sleep profiles under the standard diet (Fig. 3A). The sleep profile under the malnutrition + EAAs diet was very different from that under the standard diet (compare Fig. 3A and 3C). Whilst rhythm strength, activity and the duration of one activity period decreased upon ageing similar to observations under the standard diet, daytime sleep, the number of sleep bouts, the duration of one sleep period and the waking activity were well maintained, and night-time sleep slightly increased during ageing under the malnutrition + EAAs diet (Fig. 3C and Supplementary Fig. S2C). The waking activity was maintained during ageing under the malnutrition + EAAs diet; thus, the increased amount of sleep was not simply due to sickness of flies owing to supplementation of EAAs (Supplementary Fig. S2). The observed differences in the effects of the two diets on sleep profiles might be partly due to the higher bioavailability of free EAAs in the malnutrition + EAAs diet than in the standard diet, which contains the same level of EAAs as a protein source (yeast).

**Fig. 5:**
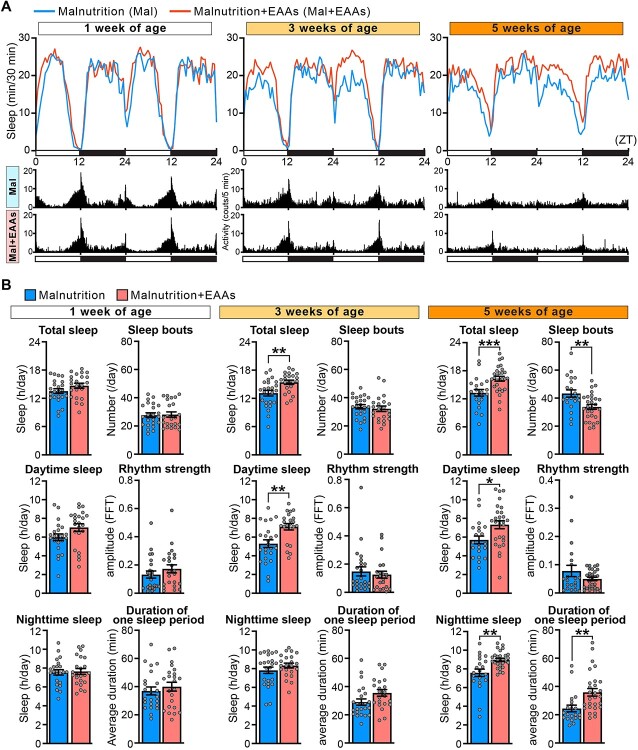
**Supplementation of the malnutrition diet with EAAs suppresses age-related sleep loss and sleep fragmentation but not loss of rhythm strength and activity.** (A) Sleep profiles and actograms from wild-type male flies maintained on the yeast-restricted malnutrition diet (Malnutrition) or the malnutrition diet supplemented with 10 EAAs (Malnutrition + EAAs) at the age of 1, 3 and 5 weeks. Average sleep amounts in the 30-min bin are plotted at ZT. Daytime and night-time under the light–dark condition is indicated in white and black bars at the bottom. The actogram shows the average number of infrared beam crossings for 5-min-bin. (B) Total sleep, daytime sleep, night-time sleep, number of sleep bouts, duration of one sleep period and rhythm strength are shown as mean ± SEM. *n* = 21–28, ^*^P < 0.05, ^**^P < 0.01 and ^***^P < 0.001 by Student’s *t*-test.

To further elucidate the effects of EAA supplementation on age-associated sleep profiles, we directly compared the two datasets under the malnutrition diet and malnutrition + EAAs diet at each age. This analysis revealed that the amounts of total sleep, daytime sleep and night-time sleep were significantly increased under the malnutrition + EAAs diet in 3- and/or 5-week-old flies (Fig. 5). In addition, the number of sleep bouts decreased and the duration of one sleep period increased upon supplementation of EAAs in 5-week-old flies (Fig. 5). Moreover, the waking activity was slightly but significantly increased under the malnutrition + EAAs diet in 5-week-old flies (Supplementary Fig. S4). As expected, EAA supplementation did not significantly alter rhythm strength (Fig. 5).

Taken together, these results indicate that EAA supplementation ameliorates age-associated sleep loss and sleep fragmentation, but not loss of rhythm strength and activity, under poor nutrition status due to a yeast-restricted malnutrition diet in *Drosophila*.

## Discussion

Sleep duration and quality decline with age, and the prevalence of sleep disorders increases in the elderly (17). Nutrition and nutritional status are closely related to sleep (26), and older adults are more likely to be malnourished due to nutrient deficiency, which may exacerbate sleep problems. The sleep-like state in *Drosophila* has many similarities to sleep in humans (37, 41) and is regulated by two independent processes: circadian rhythmicity and sleep homeostasis (48). As in mammals, the quality and quantity of sleep in *Drosophila* decline with age (38, 40, 41), suggesting that *Drosophila* is a model to investigate age-related sleep problems. Accumulation of free radicals (40), disruption of metabolism (49–51) and dysregulation of circadian clock-related neural networks (52, 53) underlie age-related alterations of sleep profiles, whilst reduced insulin-like signalling ameliorates sleep fragmentation in aged flies (54). In this study, we demonstrated that supplementation of EAAs ameliorated age-associated sleep loss and sleep fragmentation in *Drosophila*.

Several studies have investigated the effects of dietary conditions on sleep-wake behaviour in *Drosophila* (55). Starvation/fasting suppresses sleep to promote foraging behaviour in *Drosophila* (56). By contrast, a high-calorie diet (high-fat diet) accelerates age-associated sleep fragmentation accompanied by cardiac dysfunction and a shortened lifespan (49, 50). Dietary sugar also alters sleep behaviour during both the light and dark phases of the circadian period, including a change in the arousal threshold for waking, independent of circadian rhythmicity, total sleep, latency to sleep and waking activity (57). There is some discrepancy in the effects of protein on sleep-wake behaviour in *Drosophila* (57–60). Two studies reported that increasing the amount of dietary yeast, the primary source of protein in fly food, negatively impacts sleep integrity. One study reported that male flies maintained on a diet containing 2% yeast extract exhibit sleep loss and sleep fragmentation compared with those maintained on a diet containing 0% yeast extract (58). Another study showed that male flies maintained on a diet containing 20% yeast exhibit sleep loss and sleep fragmentation compared with those maintained on a diet containing 5% yeast (60). By contrast, other studies did not find significant effects of dietary yeast on sleep and wakefulness (57, 59). In this study, we demonstrated that age-associated sleep loss, sleep fragmentation and loss of rhythm strength were very similar in male flies under the standard diet containing 2.7% yeast and the malnutrition diet containing 0.27% yeast (Fig. 3, 4, Supplementary Fig. S2 and S3), except for a slight decrease in sleep in young flies under the malnutrition diet (Fig. 4). By contrast, both TOR signalling activity and lifespan were significantly lower in male flies maintained on the malnutrition diet than in those maintained on the standard diet as expected (Fig. 2). These results indicate that sleep profiles of male Canton-S flies are maintained in 0.27–2.7% dietary yeast conditions and that age-associated loss of sleep integrity does not always correlate with altered TOR activity and lifespan in male flies.

Another interesting finding of this study is that supplementation of the malnutrition diet with EAAs had complex effects on lifespan, TOR signalling activity and sleep. A previous report showed that the lifespan of flies maintained on a diet containing 20% yeast is much shorter than that of flies maintained on a diet containing 10% yeast accompanied by increased TOR activity, and this lifespan-shortening effect is explained by the high EAA content of the yeast-rich diet (46, 61, 62). By contrast, our data showed that the lifespan of flies maintained on the malnutrition diet containing 0.27% yeast was significantly shorter than that of flies maintained on the standard diet containing 2.7% yeast, and this lifespan-shortening effect was not simply explained by the low EAA content of the yeast-poor diet. Supplementation of the malnutrition diet with EAAs to the same level as the standard diet fully restored TOR signalling activity as expected (Fig. 2A); however, it did not restore the lifespan of flies (Fig. 2B). By sharp contrast, supplementation of the malnutrition diet with EAAs significantly suppressed age-associated sleep loss and sleep fragmentation without altering the loss of rhythm strength and activity (Fig. 3, 4, 5, Supplementary Fig. S2, S3 and S4). These results reinforce that loss of sleep integrity does not always correlate with increased or decreased TOR activity and longevity and suggest that sleep duration and sleep strength are determined independent of rhythm strength and locomotor activity in male flies. A previous genetic study reported that flies with short and fragmented sleep display phenotypes associated with increased ageing, as well as a shortened lifespan (63). Our results suggest that environmental factors such as dietary conditions have complex effects on lifespan, activity, sleep profiles and nutrient-sensing pathways.

Our results demonstrated that supplementation of amino acids showed more prominent rescue effects on daytime sleep than on night-time sleep (Fig. 5B, 3 weeks of age). We speculate that this is partly because daytime sleep was more affected than night-time sleep in aged Canton-S male flies and thus night-time sleep seemed to have only slightly increased by amino acids supplementation due to the ceiling effects (Fig. 5B, 3 weeks of age). The sleep profile in *Drosophila* shows a bimodal pattern with peaks at noon and midnight (Fig. 1B), and the timing of this sleep is controlled by circadian clocks. Several factors have been reported to affect daytime and night-time sleep differently (64). For example, as in mammals, light promotes arousal in *Drosophila*, the wake-promoting peptide pigment-dispersing factor (PDF) (analogous to hypocretin in mammals) is secreted by cells in the central clock network in response to light, whilst GABAergic inputs inhibit these neurons and promote sleep (65). In addition, ecdysone signalling (a steroid hormone in insects) and sex peptides (acting specifically on female flies) have been reported to affect daytime sleep (66, 67). These molecular mechanisms and/or neural circuits may be susceptible to ageing, and supplementation of amino acids under malnutrition conditions might have positive impacts on these processes.

Regarding the mechanism underlying the protective effects of EAA supplementation on sleep integrity, several studies reported that metabolism of EAAs, especially branched-chain amino acids, plays a crucial role in regulation of wakefulness/sleep (68). In a more recent study, Ki et al. systematically investigated the effects of EAAs and non-EAAs on sleep amount using *Drosophila* and reported that administration of threonine, histidine and arginine increases the amount of sleep. In particular, dietary threonine promotes sleep through GABAergic control (69). Thus, valine, leucine, isoleucine, threonine, histidine and arginine, which were among the EAAs supplemented to the malnutrition diet in this study, may help to ameliorate age-associated sleep loss and sleep fragmentation. As in mammals, dopamine, octopamine (analogous to norepinephrine) and histamine act as wake-promoting neurotransmitters, whilst GABA and serotonin act as sleep-promoting neurotransmitters in *Drosophila* (70)*.* All these neurotransmitters are synthesized from amino acids, and we speculate that the sleep-promoting neurotransmitter system may be affected in aged flies. For example, sleep-promoting serotonin is synthesized from one of the EAAs, tryptophan, and of particular interest, the administration of serotonin significantly and preferentially increases the amount of daytime sleep in *Drosophila* (71). It is also noteworthy that although supplementation of EAAs to the malnutrition diet was designed to achieve the same level of EAAs contained in yeast in the standard diet, the malnutrition + EAAs diet significantly ameliorated age-associated sleep loss and sleep fragmentation compared with the standard diet. We speculate that this is due to the higher bioavailability of EAAs in the malnutrition + EAAs diet, which may be directly and efficiently absorbed and utilized to synthesize neurotransmitters and other substances that promote sleep compared with EAAs provided as yeast in the standard diet (compare Fig. 3A and 3C).

## Conclusion

This study demonstrates that the malnutrition diet with restricted yeast, the primary source of protein, significantly decreases TOR signalling activity and the lifespan of male Canton-S flies, but has a minimal impact on alterations of sleep profiles during ageing. By sharp contrast, supplementation of this diet with EAAs significantly ameliorates age-associated sleep loss and sleep fragmentation without altering rhythm strength and activity. The observed protective effects of EAAs on sleep integrity seem to be independent of TOR signalling activity and lifespan regulation. These results may contribute to the development of dietary interventions that improve age-related sleep problems in humans.

## Supplementary data

Supplementary Data are available at *JB* Online.

## Supplementary Material

Web_Material_mvae090
