# Supplementary material for: Supplementation of essential amino acids suppresses age-associated sleep loss and sleep fragmentation but not loss of rhythm strength under yeast-restricted malnutrition in Drosophila
Source: J Biochem. 2024 Dec 19;177(3):225–37. doi: 10.1093/jb/mvae090 (PMC11879319; doi:10.1093/jb/mvae090)
Supplement: Web_Material_mvae090 [file web_material_mvae090.zip › Malnutrition supplmentary data.pdf]

# Supplementary data

**Supplementation of essential amino acids suppresses age-associated sleep loss and sleep fragmentation but not loss of rhythm strength under yeast-restricted malnutrition in *Drosophila***

Sachie Chikamatsu[1,2], Yasufumi Sakakibara[1], Kimi Takei[1], Risa Nishijima[1], Koichi M. Iijima[1,2,*], Michiko Sekiya[1,2,*]

[1]Department of Neurogenetics, Center for Development of Advanced Medicine for Dementia, National Center for Geriatrics and Gerontology, Obu, Aichi, Japan
[2]Department of Experimental Gerontology, Graduate School of Pharmaceutical Sciences, Nagoya City University, Nagoya, Aichi, Japan

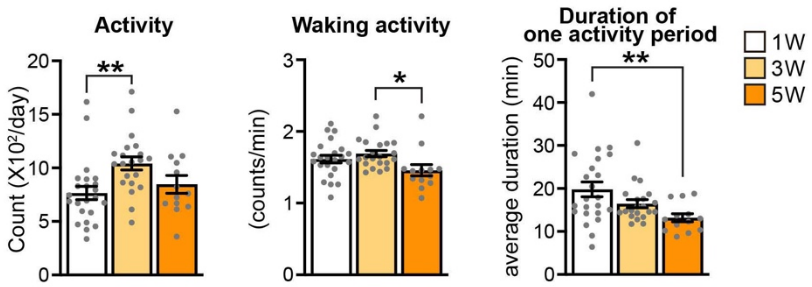


**Figure S1. Age-associated alterations of activity profiles under the standard diet in *Drosophila***

(**A**) The total activity, waking activity, and duration of one activity period from male wild type flies maintained on the standard diet at 1, 3, and 5 weeks of age are shown as mean ± SEM, n=13-23, $^{*}p < 0.05$ and $^{**}p < 0.01$ by one-way ANOVA followed by Tukey's post-hoc tests.

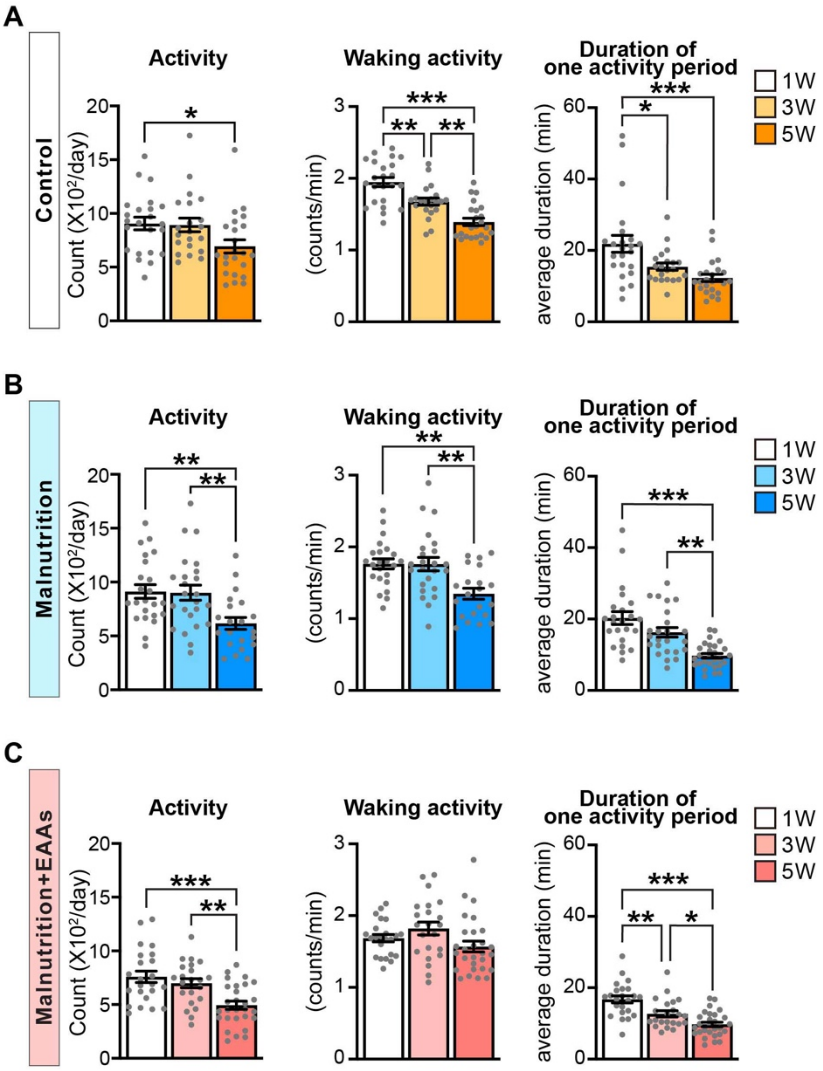


**Figure S2. Age-associated alterations of activity profiles under the standard diet, the yeast-restricted malnutrition diet, and the malnutrition diet with essential amino acids in *Drosophila***

(**A**-**C**) The total activity, waking activity, and duration of one activity period from wild type male flies maintained on the standard diet (Control) (**A**), yeast-restricted malnutrition diet (Malnutrition) (**B**), and malnutrition diet supplemented with 10 essential amino acids (Malnutrition + EAAs) (**C**) at the age of 1, 3, 5 weeks. Data are shown as mean ± SEM. n=21-24, $^{*}p < 0.05$, $^{**}p < 0.01$ and $^{***}p < 0.001$ by one-way ANOVA followed by Tukey's post-hoc tests.

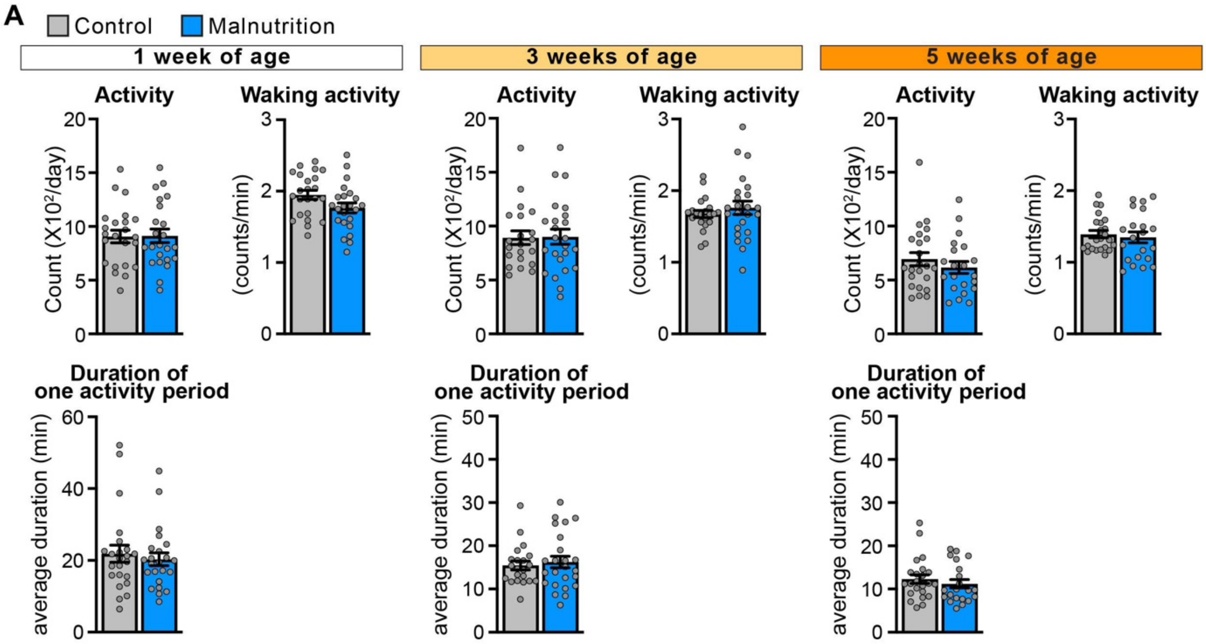


**Figure S3. Activity profiles under the standard diet and the yeast-restricted malnutrition diet in *Drosophila***

(**A**) The total activity, waking activity, and duration of one activity period from wild-type male flies maintained on the standard diet (Control) or yeast-restricted malnutrition diet (Malnutrition) at the age of 1, 3, and 5 weeks. Data are shown as mean ± SEM. n=21-28.

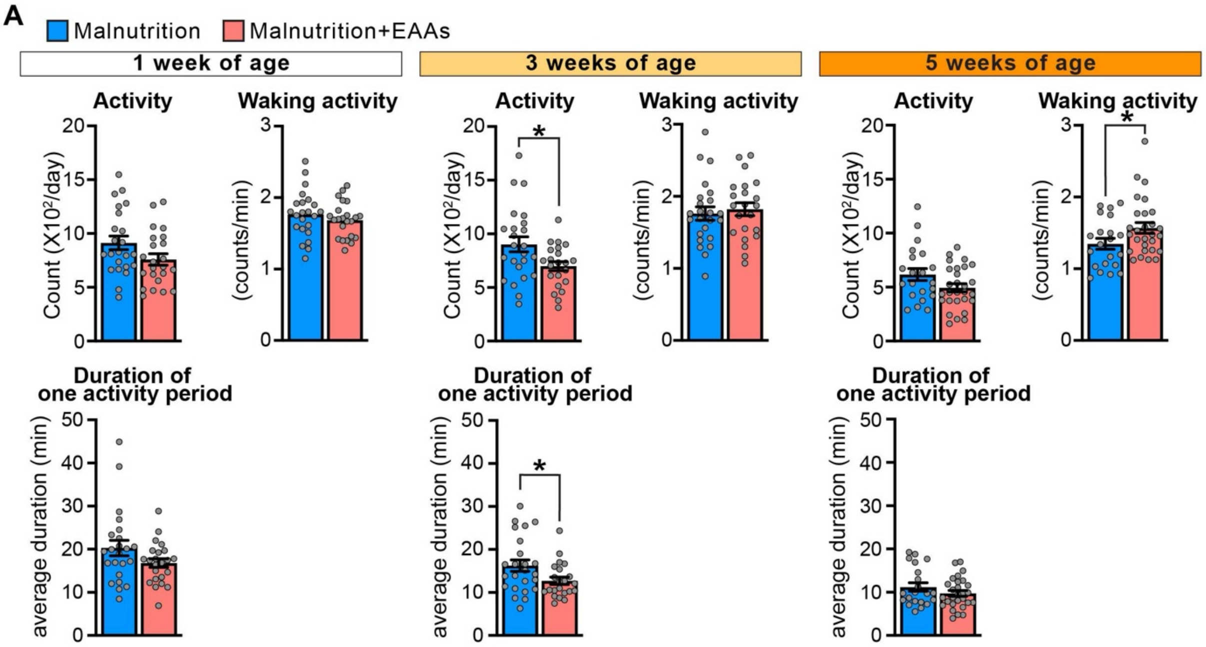


**Figure S4. Activity profiles under the yeast-restricted malnutrition diet and malnutrition diet with essential amino acids in *Drosophila***

(**A**) The total activity, waking activity, and duration of one activity period from wild-type male flies maintained on the yeast-restricted malnutrition diet (Malnutrition) or malnutrition diet supplemented with 10 essential amino acids (Malnutrition + EAAs) at the age of 1, 3, and 5 weeks. Data are shown as mean ± SEM. n=21-28, $^{*}p < 0.05$ by Student's *t*-test.
